# GWAS provides biological insights into mechanisms of the parasitic plant (*Striga*) resistance in sorghum

**DOI:** 10.1186/s12870-021-03155-7

**Published:** 2021-08-21

**Authors:** Jacinta Kavuluko, Magdaline Kibe, Irine Sugut, Willy Kibet, Joel Masanga, Sylvia Mutinda, Mark Wamalwa, Titus Magomere, Damaris Odeny, Steven Runo

**Affiliations:** 1 Department of Biochemistry, Microbiology and Biotechnology, Kenyatta University, Nairobi, Kenya; 2 Pan African University of Science Technology and Innovation, Jomo Kenyatta University of Agriculture and Technology, Nairobi, Kenya; 3 International Crops Research Institute for the Semi-Arid Tropics, Nairobi, Kenya

**Keywords:** Genome-wide association studies (GWAS), Parasitic plants, *S. hermonthica* (*Striga*), *Striga* resistance, Sorghum diversity panel

## Abstract

**Background:**

Sorghum yields in sub-Saharan Africa (SSA) are greatly reduced by parasitic plants of the genus *Striga* (witchweed). Vast global sorghum genetic diversity collections, as well as the availability of modern sequencing technologies, can be potentially harnessed to effectively manage the parasite.

**Results:**

We used laboratory assays – rhizotrons to screen a global sorghum diversity panel to identify new sources of resistance to *Striga*; determine mechanisms of resistance, and elucidate genetic loci underlying the resistance using genome-wide association studies (GWAS). New *Striga* resistant sorghum determined by the number, size and biomass of parasite attachments were identified. Resistance was by; i) mechanical barriers that blocked parasite entry, ii) elicitation of a hypersensitive reaction that interfered with parasite development, and iii) the inability of the parasite to develop vascular connections with hosts. Resistance genes underpinning the resistance corresponded with the resistance mechanisms and included pleiotropic drug resistance proteins that transport resistance molecules; xylanase inhibitors involved in cell wall fortification and hormonal regulators of resistance response, Ethylene Response Factors.

**Conclusions:**

Our findings are of fundamental importance to developing durable and broad-spectrum resistance against *Striga* and have far-reaching applications in many SSA countries where *Striga* threatens the livelihoods of millions of smallholder farmers that rely on sorghum as a food staple.

**Supplementary Information:**

The online version contains supplementary material available at 10.1186/s12870-021-03155-7.

## Background

Sorghum is a preferred staple cereal for millions of people in sub-Saharan Africa (SSA) but its production is greatly constrained by the root parasitic plant *S. hermonthica* Del. Benth.; This hemi-parasitic plant in the family Orobanchaceae attaches to roots of cereal crops, siphons their nutrients and may lead to death of the infected host. Yield losses due to *Striga* in Africa are severe and range from 30 to 100% [1] translating to an estimated USD 7 billion every year [2].

*Striga* control methods are limited. These include cultural and agronomic practices [3], seed treatment with herbicides [4], use of trap crops [5] and deployment of resistant varieties [6, 7]. Although these strategies have been used for a long time, they are either ineffective or poorly adaptable by African smallholder farmers [8]. *Striga* resistance breeding is a viable management strategy [9]. However, resistance can be short-lived and overcome by the parasite [2]. An effective resistance breeding approach will be one that combines several sources of resistance (pyramiding) but realizing such control is limited by availability of well characterised sources of resistance [10].

In characterizing *Striga* resistance sources, it is important to firstly, delineate the various mechanisms used by hosts to fight against the parasite. Broadly, these occur at two levels; (i) pre-attachment, which includes resistance mechanisms that hinder parasite germination and haustorium development [11–14] and, (ii) post-attachment, resistance mechanisms that hinder parasite attachment and growth inside host tissue – such as mechanical barriers [15], secondary metabolites [16], and hypersensitive reactions [17]. Upon determination of the causes, genetic loci underpinning such mechanisms can then be mapped. In sorghum, earlier *Striga* resistance loci mapping studies used quantitative trail loci (QTL) analysis based on simple sequence repeat and amplified fragment length polymorphism (AFLP) markers in two recombinant inbred line (RIL) populations derived from crosses between; (i) post-attachment resistant cultivar N13 and the susceptible cultivar E36–1 (ii) pre-attachment resistant IS9830 crossed to the susceptible cultivar E36–1 [18]. Subsequent studies have used genotyping by sequencing (GBS)-based single nucleotide polymorphisms (SNPs) in genome wide association studies (GWAS) to determine genomic regions of sorghum associated with adaptation to *Striga* [19]. Although these studies have provided insights to genetic/genomic regions linked to *Striga* resistance in sorghum, interpreting field GWAS studies is confounded by environmental variability that sometimes complicate interpretation of association data [18]. Moreover, it has not been possible to determine genetic mechanisms specifically responsible for post-attachment *Striga* resistance.

Here, we evaluated the hypothesis that genetic mechanisms underpinning specific post-attachment *Striga* resistance in sorghum can be resolved by GWAS and that confounding environmental variability can be lessened by carrying out *Striga* resistance assays under controlled greenhouse conditions. We determined the post-attachment resistance responses of sorghum accessions derived from a large diversity panel using rhizotron assays. To home in on genomic regions associated with *Striga* resistance at high resolution, we used GBS-based SNPs. Together, these analysis led us to unravel various genetic mechanisms that sorghum uses to battle *Striga* parasitism.

## Results

### The sorghum diversity panel is genetically structured according to races and geographical origin

**Fig. 1:**
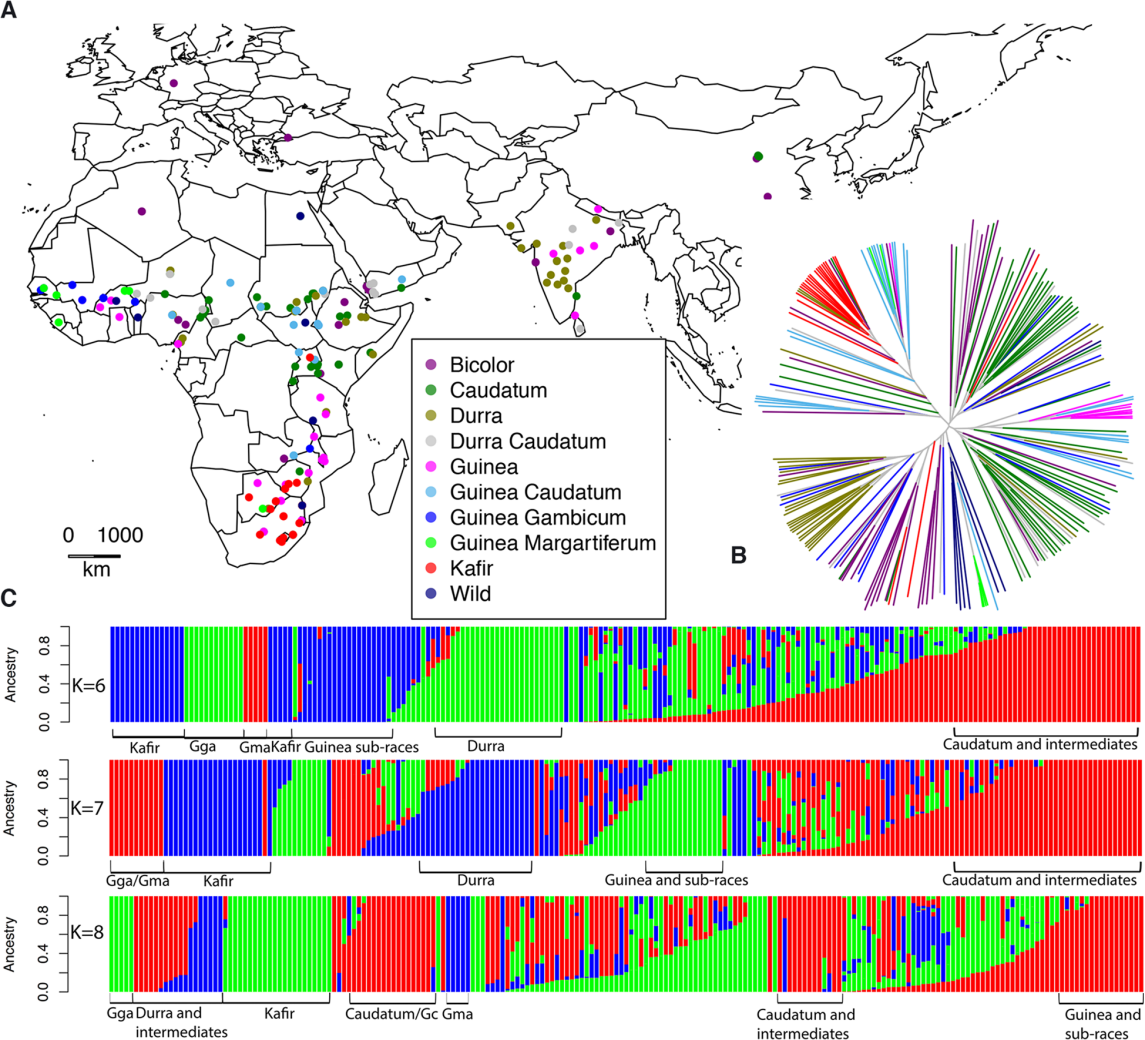
Population structuring of the sorghum diversity panel. (**A**). A distribution map showing the geographical origin of the 206 sorghum genotypes. (**B**). A phylogenetic tree of genotypes in the sorghum diversity panel according to their origin. Ancestry was inferred using the Neighbor-joining algorithm implemented in APE. Branch colors on the phylogenetic tree correspond with dots on the map to represent the basic sorghum races. (**C**). Hierarchical population structuring of the sorghum diversity panel. Stratification was performed using ADMIXTURE based on K values (ranging from 1 to 10). The indicated K values had the lowest CV values

The sorghum diversity panel constituted all major cultivated, wild and intermediate races. Distribution of these genotypes was across Africa, India, the Middle East, Europe and North America. Information on the geographical origin, general races, and population structure is outlined in Fig. 1; Fig. S1; Table S1. Caudatum and its intermediate races represented the most common genotypes in the panel (40.54%) and were found distributed across all continents. A majority of them were from Africa (East, West and Central), and clustered based on their geographic origin (Fig. 1B,C; Table S1). Kafir genotypes were mostly distributed in Southern Africa, their centre of origin. Bicolor was found in diverse geographical locations in Africa (East, South and West Africa), North America, East Asia (China), India and the Mediterranean region (Fig. 1A). Durra was mostly distributed in the Indian subcontinent and Eastern Africa, although Central Africa also showed a few Durra and the sub-race Durra muskwaari (Dmkr). Furthermore, intermediate race Durra-caudatum (DC) was predominant in India but also well represented in East Africa and the Middle East. Wild sorghum genotypes of subspecies *arundinaceum, drummondii* were distributed in West Africa but also present in West Africa was the sub-race Guinea margaritiferum (Gma). Finally, the wild subspecies *verticilliflorum* were from South Africa and North America (Fig. 1C).

ADMIXTURE and NJ (Neighbour Joining) analysis were effective in differentiating the sorghum genotypes. In ADMIXTURE, a cross-validation approach revealed that the most probable K ranged from 6 to 9 with K = 7 having the lowest CV (Cross Validation) error. This analysis led to 5 major clusters of genotypes with minor overlaps. Overall, differentiation was according to the race structures rather than geographic origins of sorghum genotypes and comprised Guinea sub-races, Kafir, Durra, Guinea and Caudatum. Although NJ was comparable to ADMIXTURE in differentiating the genotypes, it was not possible to get a distinct Caudatum cluster. Instead, we obtained clusters of Guinea, Kafir, Durra, and Guinea sub-races (Fig. 1C).

Further characterization of the population structure using principal components (PC), showed that PC1, PC2, and PC3 explained the highest proportion of the total variance. A PCA plot (Fig. S1) produced using these PCs affirmed the stratification of the sorghum diversity panel earlier described in ADMIXTURE and NJ. A total of five distinct clusters were generated with the most variant sub group consisting of Kafir (PC1), Guinea -Caudatum and Caudatums (PC2) and sub-race Guinea margaritiferum on PC3.

### Sorghum genotypes in the diversity panel exhibit different levels of post-germination resistance against *S. hermonthica*

**Fig. 2:**
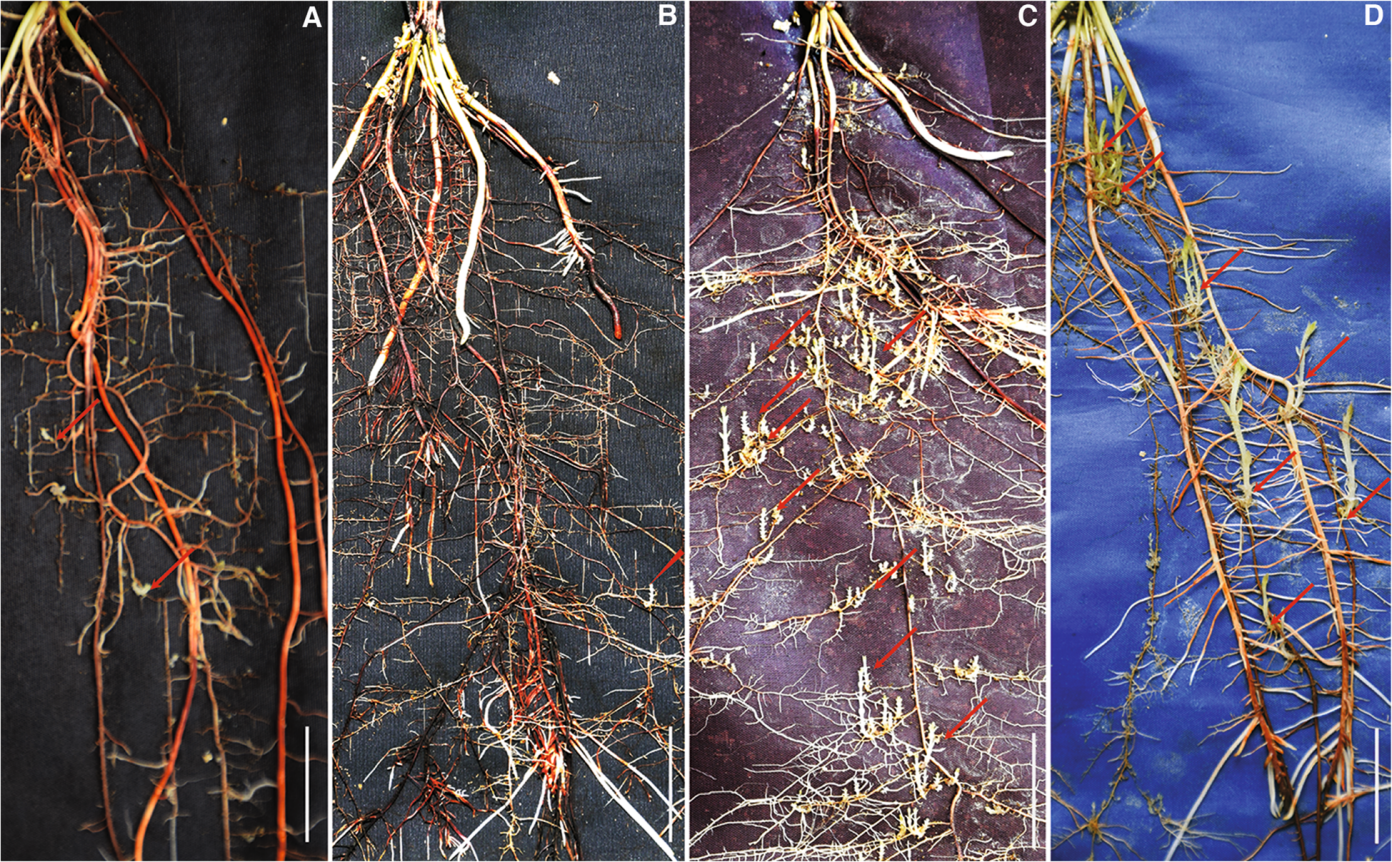
Resistance response of sorghum genotypes to *S. hermonthica* infection on rhizotrons at 21 DAI. (**A**) and (**B**) Resistant phenotypes exhibited by IS9830 and IS14963, respectively. The genotypes have short and few parasite attachments. (**C**) and (**D**) Susceptible phenotypes represented by IS20016 and IS16396, respectively. The genotypes have numerous and long attachments. Arrows indicate parasite attachments on host roots. Bar = 2 cm

Resistance response of sorghum accessions as determined using the rhizotron assay provided three metrics for measuring the levels of resistance: Mean number of *Striga* attachments, mean *Striga* length and mean *Striga* biomass. Various resistance responses based on numbers and sizes of *Striga* seedlings attached on representative host roots are shown in Fig. 2. Resistant genotypes such as IS9830 and IS14963 (Fig. 2A,B) supported few, and short parasite seedlings with low *Striga* biomass while susceptible responses for example IS20016 and IS16396 (Fig. 2C,D) showed successful parasite colonization marked by numerous and long *Striga* plants with a large biomass.

Responses were categorized as; “Highly Resistant”, “Resistant”, “Moderately Resistant”, “Susceptible” and “Highly Susceptible” based on comparisons with the resistant controls (N13 and IS9830) and the susceptible check (Ochuti). Proportions for different categories of resistance as determined by number of *Striga* attachments, *Striga* length and *Striga* biomass are presented in Figs. S2A, B and C respectively. Resistance proportions by mean number of *Striga* attachments were as follows: “Highly Resistant” = 6.8%, “Resistant” = 27.1%, “Moderately Resistant” = 14.6%, “Susceptible” = 18% and “Highly Susceptible” = 33.4% while proportions of resistance measured by mean *Striga* length were as “Highly Resistant” = 2.9%, “Resistant” = 6.8%, “Moderately Resistant” = 12.1%, “Susceptible” = 12.6% and “Highly Susceptible” = 65.5%. And resistance proportions determined using mean *Striga* biomass were as follows: “Highly Resistant” = 18%, “Resistant” = 27.7%, “Moderately Resistant”, “Resistant” = 20.9%, “Susceptible” = 11.1% and “Highly Susceptible” = 22.8%. To better understand the metric that contributed the most to overall variation in resistance among the sorghum genotypes, the 3 metrics were subjected to PC analysis (Fig. S2). Results showed that number of *Striga* attachments and *Striga* biomass accounted for most (89.0%) of the total variance distributed. Particularly, number of *Striga* attachments accounted for 61.2%, while *Striga* biomass and *Striga* length accounted for 27.8 and 11.0% of the variance, respectively (Fig. S2D).

**Fig. 3:**
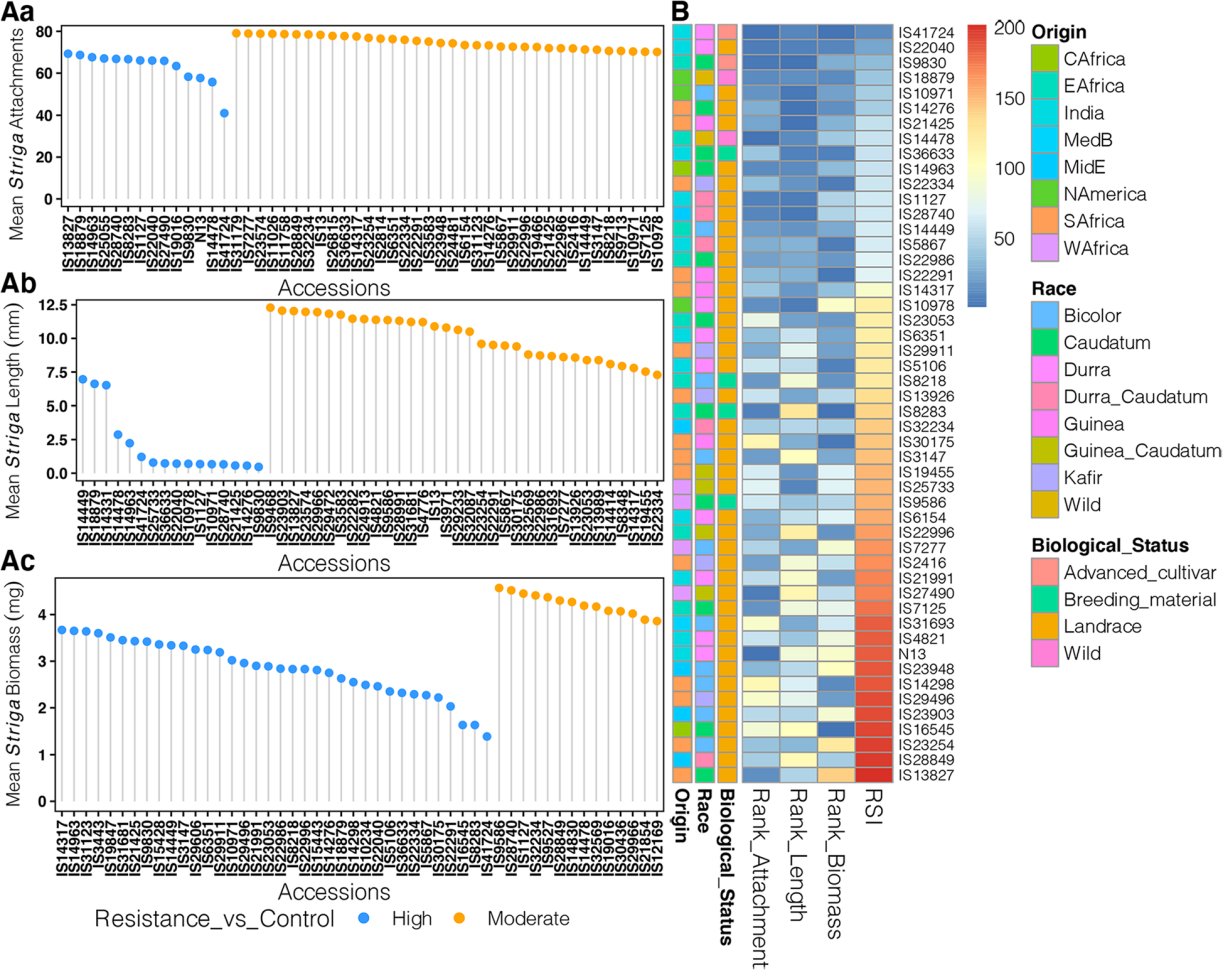
Analysis of *Striga* post-attachment resistance. (**Aa**) Dot plot of *Striga* mean attachments for 50 accessions with the lowest number of *Striga* attachments. (**Ab**) Dot plot of *Striga* mean length for 50 accessions with the lowest *Striga* length and (**Ac**) Dot plot of *Striga* mean biomass for 50 accessions with the lowest *Striga* biomass. Resistance categories are color coded based on resistance relative to resistant checks, either N13 or IS9830: blue = similar to or higher than resistant controls “Highly Resistant”, yellow = one mean separation lower than resistant controls “Resistant”. (**B**) Heatmap the top 50 most resistant accessions based on ranked summation index (RSI) of *Striga* attachments, *Striga* length, and *Striga* biomass. Annotations for origin, race and biological status of the accessions are also shown

Next, we considered individual resistance responses of the sorghum accessions. These results are presented in Table S2. Additionally, Fig. 3 is a representation of the 50 best performing accessions as determined by number of *Striga* attachments (Fig. 3Aa), *Striga* length (Fig. 3Ab) and *Striga* biomass (Fig. 3Ac).

*Striga* resistance, as measured by number of seedlings attached on sorghum genotypes, ranged from 41.00 ± 0.53 in the most resistant genotype (IS41724), to 292.70 ± 0.26 for the most susceptible (IS20016), a Guinea gambicum landrace from West Africa. Noteworthy, IS41724 and IS14478, a wild drummondii from East Africa had significantly lower number of attachments than either of the resistant controls IS9830, (number of attachments = 58.27 ± 0.61) and N13, (number of attachments = 57.67 ± 1.10). Ten other genotypes were categorized as highly resistant because the number of attachments on their roots were not significantly different from those of IS9830. By this metric, the susceptible check Ochuti (number of attachments = 116.60 ± 0.79) was categorized as “Highly Susceptible”.

Based on *Striga* mean length, the most resistant genotype was again IS9830 (mean length of *Striga* attachments = 0.47 ± 0.08 mm), while the most susceptible one was IS9168 a Kenyan Bicolor landrace (mean length of attachments = 72.96 ± 0.70 mm). The length of IS9830 was not significantly different from 15 other sorghum genotype and these formed the “Highly Resistant” category. N13, supported relatively larger seedlings (15.72 ± 0.19 mm) and fell into the category of “Moderately Resistant”. In comparison, the susceptible check Ochuti had seedlings measuring 47.93 ± 0.58 mm and was categorized as “Highly Susceptible”.

With regard to mean weight of *Striga*, resistance ranged from 1.39 ± 0.04 l mg in IS41724 to 32.97 ± 0.22 mg in SSM276, which is a landrace of a Guinea margaritiferum sub-race from Burkina Faso. The biomass of the most resistant genotypes (IS41724) was statistically similar to that of IS9830 (mean *Striga* biomass = 3.42 ± 0.12 mg) and 34 other genotypes. “The Highly Susceptible” category had 46 genotypes which also included the susceptible control, Ochuti, had a mean biomass of 11.77 ± 0.77 mg.

In general, genotypes with short seedlings also had few attachments and therefore a correspondingly low biomass. However, in some genotypes, the *Striga* mean length was low but the attachments were numerous leading to a large biomass. For example, IS22239 was placed in the “Highly Susceptible” category using number of attachments (205.73 ± 14.88) but in the “Resistant” category using mean *Striga* length (12.31 ± 2.70 mm). Its biomass was 28.03 ± 1.17 mg which was in the “Highly Susceptible” category. In other cases, genotypes had long but few attachments. For example, the genotype N13 had relatively long (15.72 ± 0.19 mm) but few (57.67 ± 1.10) attachments and consequently a moderate biomass (6.33 ± 0.11 mg).

The Rank Summation Index (RSI) [11, 20] was computed to determine the combined resistance responses as measured by *Striga* attachments, *Striga* length and *Striga* biomass. These results are presented in Fig. 3B as a heatmap for the top 50 accessions and in Table S3. Further, a list of the top 10 resistant accessions is presented in Table 1. Among the genotypes only IS9830 had previously been reported as resistant [18]. To our knowledge, the resistance of the other 9 genotypes have never been described and form a new genetic resource for *Striga* resistance improvement in sorghum. We would like to highlight two genotypes because of their advanced breeding status and therefore high potential for immediate integration in *Striga* resistance programs in SSA. Firstly, the genotype IS41724, an advanced Indian Durra breeding line which ranked highest with regard to fewest number of *Striga* attachments (41.00 ± 0.53); least *Striga* length (1.21 ± 0.10 mm) and least *Striga* biomass (1.39 ± 0.04 mg) and secondly, IS36633 also an Indian breeding germplasm of the Caudatum race with resistance metrics of *Striga* attachments (77.73 ± 1.21) *Striga* length (0.73 ± 0.04 mm) *Striga* biomass (2.32 ± 0.17 mg). Also noteworthy, in this list of resistant genotypes are two wild sorghum accessions IS18879, a wild Arundinaceum from USA with metrics of resistance as follows: *Striga* attachments, (68.67 ± 1.45) *Striga* length (6.62 ± 0.44 mm) and *Striga* biomass (2.63 ± 0.09 mm) and IS14478, a wild Drummondii from Sudan with resistance metrics as follows: *Striga* attachments (55.80 ± 3.29), *Striga* length (2.87 ± 1.34 mm), and *Striga* biomass (4.19 ± 0.08 mg). The remaining genotypes were landraces sourced from Malawi (IS21425); USA; (IS10971) and India (IS22040) and South Africa (IS14276).

**Table 1:** The 10 most resistant accessions based on all metrics of attachments, length and biomass as determined by the Rank Summation Index (RSI) method

RSI rank	Genotype	Attachments	Length (mm)	Biomass (mg)	Biological status	Race	Country of origin
1.	IS41724	41.00 ± 0.53	1.21 ± 0.10	1.39 ± 0.04	Advanced cultivar	Durra	India
2.	IS22040	66.00 ± 1.11	0.71 ± 0.03	2.46 ± 0.11	Landrace	Durra	India
3.	IS9830	58.27 ± 0.61	0.47 ± 0.08	3.42 ± 0.12	Advanced cultivar	Caudatum	Sudan
4.	IS18879	68.67 ± 1.45	6.62 ± 0.44	2.63 ± 0.09	wild	Arundinaceum	USA
5.	IS10971	70.33 ± 0.81	0.67 ± 0.14	3.02 ± 0.11	Landrace	Bicolor	USA
6.	IS14276	73.27 ± 0.83	0.56 ± 0.14	2.75 ± 0.24	Landrace	Caudatum	South Africa
7.	IS21425	71.93 ± 1.15	0.57 ± 0.01	3.43 ± 0.09	Landrace	Guinea	Malawi
8.	IS14478	55.80 ± 3.29	2.87 ± 1.34	4.19 ± 0.08	wild	Drummondii	Sudan
9.	IS36633	77.73 ± 1.21	0.73 ± 0.04	2.32 ± 0.17	Breeding material	Caudatum	India
10.	IS14963	67.6 ± 0.8	2.23 ± 0.08	3.65 ± 0.17	Landrace	Caudatum	Cameroon

### Sorghum genotypes exhibit diverse mechanisms of resistance against *S. hermonthica*

**Fig. 4:**
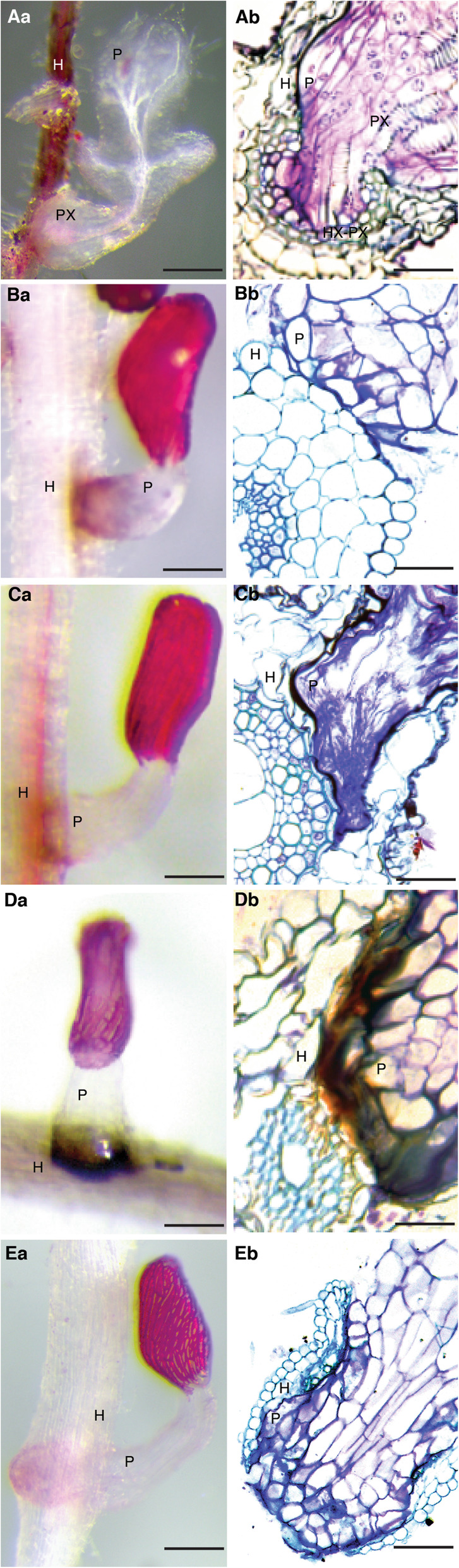
Resistance mechanisms of sorghum to *S. hermonthica* 9 DAI. (**Aa**) Colonization of IS18829 showing a well-established haustorium at the point of attachment*.* The parasite is also fully vegetative and established vascular connections with the host. Scale = 0.5 cm. (**Ab**) Transverse section through IS18829 showing penetration of the root cortex and endodermis. Penetration resulted in vascular connection between the host and parasite xylem. Scale = 0.1 mm. H = host, *P =* parasite, Hx = host xylem, Px = parasite xylem, Hx-Px = host xylem connected to parasite xylem. (**Aa**) Colonization of N13 by *Striga* showing a poorly established parasite with a darkly stained haustorium, possible because of dead cells. Scale = 1 mm. (**Bb**) Transverse section through N13 tissue showing failure of the parasite to breach the host’s cortex. Scale = 0.1 mm. (**Ca**) Colonization of IS1097810978 showing a poorly developed parasite without vascular connection. Scale = 1 mm. (**Cb**) A transverse section through an embedded IS1097810978 root tissue showing successful penetration of the cortex but parasite blockage at the endodermis. Scale = 0.1 mm. (**Da**) Colonization of IS14963 showing an intense Hypersensitive Reaction (HR) at the site of parasite infection. Scale = 1 mm. (**Db**) A transverse section through an embedded IS14963 root tissue showing blockage of the parasite tissue due to HR at the host-parasite interphase. Scale = 0.1 mm. (**Ea**) Colonization of IS9830 showing the parasite penetrating host tissue but failing to establish vascular connections. The parasite instead emerges on the opposite side of the root. Scale = 1 mm. (**Eb**) A transverse section through an embedded tissue of IS9830 showing parasite penetration of the host root and subsequent emergence in the opposite direction. Scale = 0.1 mm

Interactions between various sorghum hosts and *S. hermonthica*, are represented as close-up images between individual *Striga* seedlings attached to sorghum roots at 9 days after infection (DAI) as well as transverse sections through such haustoria (Fig. 4).

In susceptible interactions, the parasite was able to penetrate host cells and connect its vascular tissue (xylem) to that of the host to form a conduit for transfer of water and nutrients. This is the hallmark of a well-developed haustorium and subsequent proliferation of vegetative tissue, and starts with the bursting of the seed coat. This phenomenon was demonstrated in the interaction between IS18829 and *S. hermonthica*, in which we observed a well-formed haustorium with clear vascular connections between host and the parasite (Fig. 4Aa). In addition, the parasite grew rapidly and formed the five scale leaves – typical of susceptible interactions at this stage of development. A transverse section through the point of attachment indicated complete parasitic penetration of host endodermis, formation of clear xylem-to-xylem connections, as well as development of a hyaline body; a vital storage tissue for the parasite (Fig. 4Ab).

In contrast, resistant genotypes blocked parasite development at various levels by diverse mechanisms. We observed the following resistance responses; i) mechanical barrier-like that blocked parasite entry into host tissue; ii) hypersensitive reaction (HR) at the host-parasite interface, and iii) inability of the parasite to form vascular connections with the host.

#### Resistance due to mechanical barriers

In N13, parasite development was blocked before it entered the host cortex. The haustorium did not develop fully and a majority of infecting parasites did not proceed to form vegetative tissue (Fig. 4Ba). Histological analysis showed that the parasite was poorly developed (Fig. 4Bb). In total, 50% of sectioned tissue (i.e. 5 out of 10 blocks sectioned) showed this phenotype. In genotype IS1097810978, the parasite was able to penetrate the host cortex, but failed to breach the endodermis (Fig. 4Ca, Cb). Histological sections showed a parasite that went round the host pericycle instead of penetrating it (Fig. 4Cb). This resistance is suggestive of cell wall fortification or lignin deposition. The frequency of this phenotype, as determined by histological analysis, was 40% (i.e. 4 out of 10 blocks sectioned).

#### Resistance due to hypersensitive reaction

IS14963 mounted a resistance response against *Striga* characterized by an intense hypersensitive reaction at the host-parasite interface (Fig. 4Da, Db). In this genotype, a majority of seedlings (90%; *n* = 10) failed to successfully penetrate the host tissue and did not form vascular connections. Consequently, vegetative tissue failed to develop with only an intact seed coat seen during the screening period (Fig. 4Da). A section through the haustorium of IS14963 further showed clear inhibition of the parasite at the host endodermis due to necrosis (Fig. 4Db).

#### Resistance due to inability of the parasite to differentiate and form vascular connections

In the highly resistant IS9830, we observed a resistance response that occurred because the parasite was unable to differentiate and form vascular connections with the host (Fig. 4Ea, Eb). In this resistance response, the parasite attached, grew past the cortex, endodermis and emerged at the opposite end without attempting to form vascular connections with the host (Fig. 4Ea). Histological analysis confirmed failure of the parasite to differentiate and form connections with the host in most tissues (60%; *n* = 10) sectioned (Fig. 4Ea).

### GWAS identifies genetic loci associated with *S. hermonthica* in sorghum

**Fig. 5:**
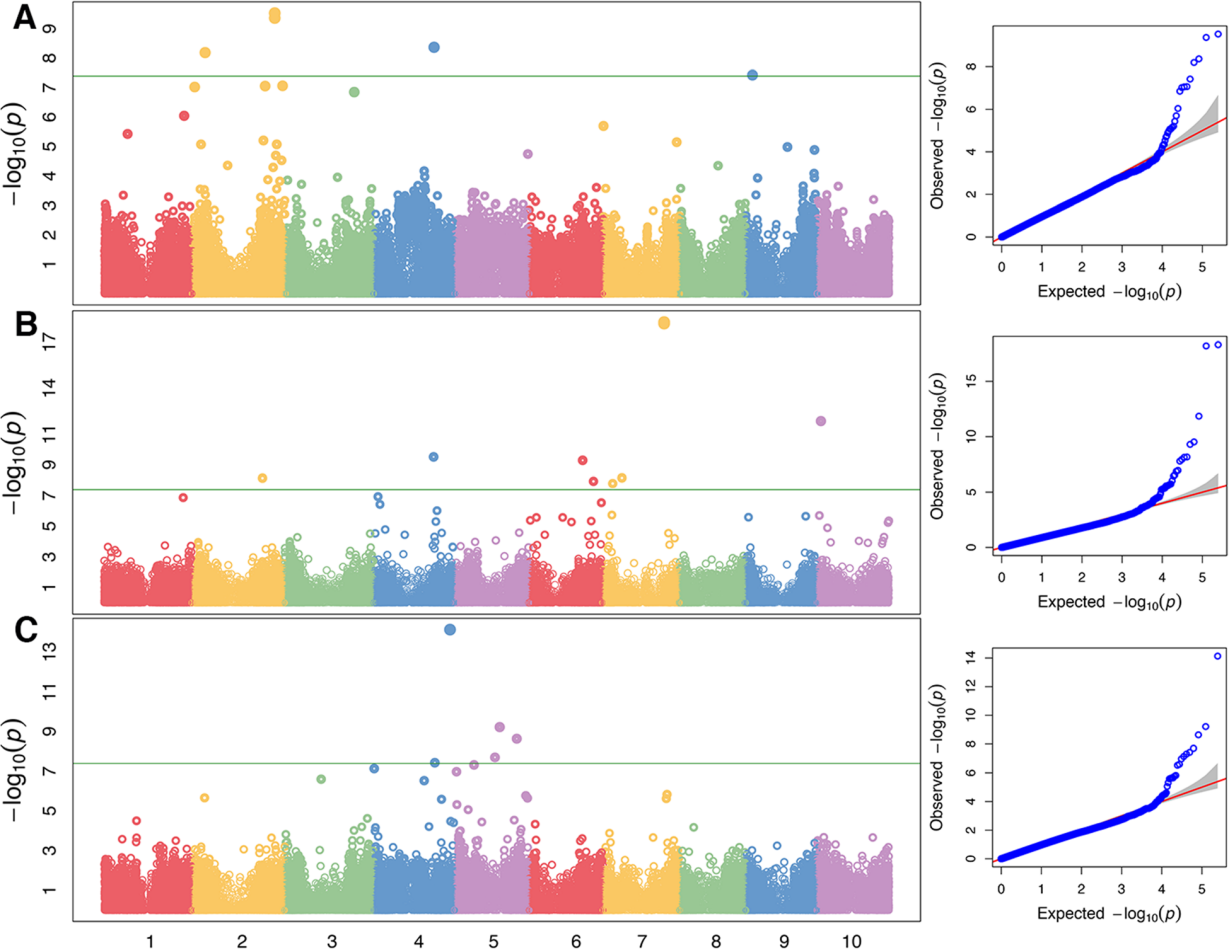
Manhattan plots of the genome-wide association studies performed using *Striga* mean number of attachments (**A**) mean length (**B**) and mean biomass (**C**). The green lines indicate the *p*-value threshold obtained using the false discovery rate (adjusted *p* < 0.05) in the FarmCPU model. Quantile-quantile plots corresponding to the Manhattan plots are presented on the left panel with the horizontal axis showing log10-transformed expected *P* values, and the vertical axis indicating log10-transformed observed *P* values

Fixed and random model Circulating Probability Unification (FarmCPU) model analysis of the sorghum diversity panel led to identification of candidate genetic regions associated with *Striga* resistance. These data are presented as Manhattan plots in Fig. 5. Suitability of the Farm CPU model for our analysis is shown using Quantile-Quantile (QQ) plots in Fig. 5. Overall, genetic causes of *Striga* resistance could be linked to observed or previously described resistance phenotypes. We found significantly associated SNPs on genes involved in the following functions: i) secretion of defense molecules, ii) modification of the host cell wall or, iii) activation of sorghum’s pathogen-mediated resistance genes. These results are summarized in Table 2.

**Table 2:** Sorghum SNPs showing significant genome-wide associations with *S. hermonthica* resistance determined using traits of mean number of attachments, mean length and mean biomass (FDR correction at ∝0.05). SNPs within genes are presented with their annotations in order of chromosomal location. AF: allele frequency; AdjP: *P*-value after FDR adjustment. *represent SNPs common to QTL detected in Haussmann et al., 2004). Letters after SNP positions represent trait to detect the SNP: a = attachment, b = length, c = biomass

Chr	Position	AF	AdjP	Gene ID	Annotation
1	66423166c*	0.25	3.03E-03	Sobic.001G375900	Hypothetical protein
1	66995004a*	0.38	2.20E-02	Sobic.001G382000	Serine/threonine-protein kinase AtPK19
2	1990072a*	0.5	3.00E-03	Sobic.002G021700	F-box proteins
2	10791041a*	0.17	3.99E-04	N/A	N/A
2	61226505a*	0.18	2.90E-03	Sobic.002G220700	Uncharacterized protein
2	75881569a*	0.14	2.90E-03	N/A	N/A
2	69292723a*	0.17	5.29E-05	Sobic.002G321300	ABCG transporter
2	59157949b*	0.46	2.52E-04	Sobic.002G201600	PMT2 Methyltransferase
3	58064948a	0.13	4.00E-03	Sobic.003G241300	Isoflavon reductase
4	3830076b	0.09	2.83E-03	obic.004G047001	NA
4	50695987a	0.31	4.00E-04	N/A	Ethylene responsive element binding factor 4
4	52466133b	0.45	5.12E-02	N/A	Uncharacterized protein
4	64068558c	0.1	2.00E-05	Sobic.004G020200	Cytosine-5- -Methyltransferase 3-Related
4	51292838c	0.14	1.84E-03	Sobic.004G163700	N/A
4	53412080b	0.43	1.69E-02	Sobic.004G181500	DNA repair protein
4	438979c	0.43	2.57E-03	Sobic.004G005100	Zinc finger with peptidase domain protein
4	5610661b	0.13	6.98E-03	Sobic.004G068800	N/A
4	50512606b	0.17	1.89E-05	Sobic.004G158901	Ethylene-responsive transcription factor ERF113
5	1591316c	0.12	3.28E-03	Sobic.005G017700	Hypothetical protein
5	33845800c	0.07	1.00E-03	N/A	N/A
5	37742821c	0.06	7.48E-05	N/A	N/A
5	52091605c	0.13	1.92E-04	N/A	N/A
5	16194394c	0.27	1.96E-03	Sobic.005G099000	Xylanase inhibitor 1
6	6412692b	0.25	3.31E-02	N/A	N/A
6	28486530b	0.38	3.31E-02	N/A	N/A
6	35866190b	0.48	5.20E-02	N/A	N/A
6	45280156b	0.16	2.46E-05	N/A	Hypothetical protein
6	52535510b	0.24	4.94E-02	N/A	N/A
6	60968111b	0.29	5.85E-03	N/A	Secondary wall NAC transcription factor 4
6	1389246b	0.33	4.90E-02	Sobic.006G009400	Peroxiredoxin1 (Prx1)
6	54417370b	0.2	3.54E-04	Sobic.006G190000	Downy mildew resistant 6 (DMR6)
7	413066a	0.12	4.40E-02	Sobic.007G004500	HSP60
7	7777464b	0.24	2.99E-02	Sobic.007G070000	Uncharacterized protein
7	8351776b	0.22	4.50E-04	N/A	N/A
7	16156498b	0.08	2.52E-04	N/A	N/A
7	51452375b	0.13	7.49E-14	N/A	N/A
7	51462880b	0.18	7.49E-14	N/A	N/A
9	5732771a*	0.39	1.80E-03	Sobic.009G056400	Fasciclin-like arabinogalactan protein 11
10	2576197b	0.19	3.02E-02	Sobic.010G032000	Early nodulin 93
10	3821956b*	0.07	1.15E-07	Sobic.010G049100	Acyl coa oxidase 1

Significant associations of *Striga* resistance with a pleiotropic drug resistance (PDR)/ ATP Binding Cassette (ABC) class G transporter at position S2_69292723 (*p =* (5.29E-05) as well as an Isoflavon reductase (IFR) at S3_58064948 (*p* = 4.00E-03) point to the importance of synthesis and secretion of secondary metabolites in defense. ABC transporters have a cytosolic nucleotide binding domain that bind ATP and a hydrolysis hydrophobic trans-membrane domain that translocate pathogen defense molecules while IFR is involved in the biosynthetic pathway of isoflavonoid phytoalexin. Interestingly, PDR transporters are known to bind and secrete phytoalexins.

We detected the Fasciclin-like arabinogalactan protein 11 that regulates plasticity and integrity of cell walls at position S9_5732771 (*p =* 1.80E-03). In addition, we found secondary cell wall modification genes for lignin biosynthesis genes: i) PMT2 Methyltransferase at S2_59157949 (*p* = 2.52E-04), ii) Secondary wall NAC transcription factor 4 at S6_60968111 (*p* = 5.85E-03) and, iii) Early nodulin 93 at S10_2576197 (3.02E-02). Finally, we detected significant associations at S5_16194394 (*p* = 1.96E-03) with Xylanase inhibitor 1 that possibly functions to inhibit *Striga*’s xylanases.

Our GWAS also revealed association with the Ethylene-responsive transcription factor ERF113 at S4_50512606 (1.89E-05). ERF113 is a key regulator of both jasmonic acid (JA) as well as salicylic acid (SA) mediated defense pathways in plants. In addition, Peroxisomal acyl-CoA oxidase 1 detected at S10_3821956 (*p* = 1.15E-07) is a key enzyme in JA biosynthesis. Downstream of defense pathway, we detected the Downy mildew resistance 6 (DMR6) at position S6_54417370b (*p* = 3.54E-04) and the hypersensitive reaction (HR) associated Peroxiredoxin1 (Prx1) at S6_1389246 (*p* = 4.90E-02). Other genes that could also putatively have a role in the innate defense pathway were a zinc finger with peptidase domain identified at S4_438979 (*p* = 2.57E-03) and Heat shock protein 60 (HSP60) at S7_ 413,066 (*p* = 4.40E-02).

The rest of the significant SNPs occurred on non-coding regions, or on genetic regions where the gene’s annotated function could not be related to pathogen resistance (Table 2).

## Discussion

Our goal was to develop a technology platform for effectively exploiting the genetic diversity of sorghum for resistance against *Striga*, a parasitic plant that greatly limits cereal production in most parts of sub-Saharan Africa. We first screened a large collection of diverse sorghum genotypes, using an efficient high-throughput *Striga* screening method based on soil-free root observation chambers called rhizotrons, in order to identify new sources of *Striga* resistance. Secondly, we characterized the mechanisms of such resistance, using detailed microscopic and histological analyses following *Striga* infection, exhibited by the sorghum genotypes. Finally, we determined the underlying genetic factors related to the resistance displayed by various sorghum genotypes using GWAS. The diversity panel provided a good resource for resistance screening and GWAS. It is reasonable to assume that variations could exist between genotyped and phenotype accessions – even with the high degree of self-fertilization in sorghum. However, such bias is greatly reduced when a large number of technical and biological replicates are used. In this study, we used 5 individuals in three replicates.

### New sources of *Striga* resistance from the sorghum diversity panel

Our findings underscore the need to continuously screen hosts for *Striga* resistance as affirmed by successful identification of new *Striga* resistant sorghum genotypes. The resistance by the genotypes, as determined by the number, size and biomass of *Striga* seedlings attached, is comparable to what has been reported in previous work that used rhizotrons [16, 21]. For example, we found an average of 57 attachments on N13 compared to a mean of 56 reported by [16] and 75 [22]. In a resistant rice variety (nipponbare) and maize (KSTP’94), parasite attachments averaged 30 [21] and 44 [23] respectively.

Overall, the rhizotron assay proved effective in screening the large number of sorghum lines for post-germination resistance to *Striga*. The metrics of resistance, in general correlated with each other. One can deduce that reduced number of attachments implies some pre-attachment mechanism while small parasites implies post-attachment mechanisms.

Rhizotron assays allowed us to identify a set of highly resistant sorghum genotypes that had considerably lower number of *Striga* attachments, length and biomass than known resistant controls. Some of these genotypes although found to be highly resistant to *Striga* are not grown in SSA. For example the Indian genotypes IS41724 (Advanced cultivar) and IS36633 (Breeding material) are good candidates for performance evaluation under *Striga* infestation in African agro-ecologies. Similarly, *Striga* resistant landraces IS21425 (Malawi); IS10971 (USA); IS14276 (South Africa) and IS22040 (India) can be evaluated for adaptability under *Striga* infestation. Finally, the resistant wild sorghum accessions IS18879 (USA) and IS14478 (Sudan) can be used a resistance source in genetic studies. Strikingly, out of a total of 206 sorghum accessions comprising 7 wild genotypes, 2 of wild sorghum accessions ranked among the best 10 performers. This underscores the importance of wild sorghum genotypes as a reservoir of disease resistance genes. Overall, integrating the *Striga* resistant varieties presented here in SSA breeding programs could complement *Striga* control efforts and contribute towards increasing yields due to reduced *Striga* infestation.

### Multiple mechanisms of *Striga* resistance

We found that sorghum uses at least three resistance mechanisms to overcome *Striga* infection. Firstly, the physical barriers that successfully stop parasite ingression into host cells at either the cortex or the endodermis. Physical barrier resistance due to thickened cell walls and lignification, are well characterised in many *Striga*-host interactions and especially in N13; a resistant Indian Durra sorghum [15]. In the current study, we observed that IS10978 also exhibited this form of resistance. In rice, such a resistance mechanism was reported in some of the New Rice for Africa (NERICA) varieties [21]. Secondly, we observed an intense hypersensitive reaction at the host-parasite interface in IS14963. This response, reminiscent of gene-for-gene resistance described in the resistant cowpea variety (b301) against the hyper virulent *S. gesnerioides* race 3 from Niger (*SG3*) [24]. In sorghum, HR-kind-of resistance was described in the resistant genotypes Dobbs, Framida, and a wild sorghum genotype P47121 [17]. Finally, we observed a new resistance mechanism displayed by IS9830, in which *Striga* was unable to differentiate and form xylem vessels. Rather, the parasite went through the host root and exited without any attempt to make connections. This phenotype was only observed in IS9830. To our knowledge, such resistance has never been reported in any *Striga*-host interactions and although the molecular and physiological mechanisms underpinning this resistance are out of the scope of the current study, inability of parasite cells to differentiate and form vascular connections with the host appears to suggest that *Striga* is insensitive to the host’s vascular differentiation signals.

### Genetic causes of *Striga* resistance

Genetic loci associated with resistance corresponded with some *Striga* resistance quantitative trait loci (QTL) reported in [18], where 2 recombinant inbred lines (RILs) each based on pre-germination resistance (IS9830) and post-germination resistance (N13) parents were used to reveal 5 QTL each associated with *Striga* resistance. In our GWAS analysis, there were overlaps in QTL from both mapping populations on chromosomes 1, 2, 9 and 10. Interestingly QTL from the IS9830 RIL overlapped with significant SNPs in our GWAS analysis even though the genotype was used as a donor for pre-germination resistance. This observation reaffirms the hypothesis that IS9830 harbours both pre-germination and post-germination resistance as previously suggested [18].

In addition to QTL mapping, a recent study [19], performed genome-wide tests of association with predicted parasite habitat suitability (HS) in 2070 sorghum landraces and found 97 genomic regions associated with *S. hermonthica* resistance. There were no common SNPs between the current study and [19], plausibly because HS scores rely on *Striga* habitat distribution factors – which can be too numerous to obscure specific components of post-germination resistance. Nonetheless general genes encoding similar mechanisms in cell wall modification, for example lignin biosynthesis were significantly associated with *Striga* resistance in both studies.

Based on significant associations in annotated genes, our study pointed to genetic processes leading to: i) synthesis and transport of secondary metabolites, ii) cell-wall modification and iii) activation of innate immunity.

We observed significant associations with ABCG/PDR transporter [25] as well as Isoflavon reductase [26] both of which are involved in secondary metabolites production and transport. In *Striga*, the PDR transporter was found to be significantly up regulated in the resistant rice cultivar (Nipponbare) following *S. hermonthica* infection [27]. Noteworthy, phytoalexins are primarily produced in leguminous plants [26] a fact that may explain their role non host incompatibility [28].

Our study also revealed Fasciclin-like arabinogalactan protein 11 involved in cell adhesion that form physical barriers against pathogen invasion [29] as well as secondary cell walls fortification encoding genes that use lignin deposition i.e. O-Methyltransferase (PMT) [30] and NAC domain transcription factor [31]. Both of these genes are involved in lignin biosynthesis and regulation, consistent with numerous studies that describe lignin as an important component in *Striga* resistance [32]. In addition to cell wall fortification, hosts may protect themselves against parasitic cell wall degrading enzymes such as a pectinesterases using their cognate inhibitors [19, 33]. In this regard, our study revealed a gene encoding xylanase inhibitor 1. Possibly, this gene encodes a *Striga* xylanase inhibitor consistent with the gene’s up-regulation in *Striga*-rice interactions [34].

Finally, consistent with the ‘zigzag’ model for *Striga*-host interactions [35], we identified genes encoding different components of pathogen activated immunity including: i) DNA repair and peroxidases; ii) DMR6 that participates in salicylic acid homeostasis and required for susceptibility to downy mildew in Arabidopsis [36] and *P. infestans* potato [37]; iii) and genes involved in induction of the Systemic Acquired Resistance (SAR) pathway which in *Striga-*rice interactions is regulated by both jasmonic acid (JA) and salycilic acid (SA) in a cross talk mediated by WRKY45 [38] and regulated by (AP2/ERFs) [39]. Interestingly, AP2/ERFs were found to be significantly associated with *S. hermonthica* resistance in white [40] and yellow tropical maize [41]. These findings underscore the importance of this pathway in *Striga* resistance.

Although most of the SNPs identified can be implicated with *Striga* resistance, it is worth noting that in some cases, there were large genetic differences between SNPs and actual genes controlling the traits. Future studies should therefore investigate the identified genes in contrasting germplasm. Nonetheless, our work has paved the way for more targeted studies and even possible breeding targets.

In summary, sorghum harbors varied mechanisms of resistance to *Striga*. The genetic factors (loci) underpinning such mechanisms are distributed within the vast sorghum gene pool of wild and cultivated genotypes. This species “richness” – genetic diversity provides an important resource that should be exploited in future *Striga* resistance breeding programs.

## Conclusions

Taken together, we report on new sources of *Striga* resistant sorghum obtained from a diverse collection. We further elucidated mechanisms of post-attachment resistance in *Striga*. Our studies employed a controlled screening assay based on rhizotrons to obtain *Striga* resistance data that was subsequently used in GWAS. This assay provided a distinct advantage over previously used methods because it provided the opportunity to control for confounding environmental variability. We were thus able to in our GWAS and successfully identified gene regions that were significantly associated with *Striga* resistance. The method is widely applicable in post-attachment resistance screening of other *Striga*-host interactions. Our study also led to identification of new *Striga* resistant sorghum varieties that can be directly integrated in sorghum improvement programs in SSA. Moreover, elucidation of new mechanisms of resistance will allow breeders to develop material with multiple forms of resistance for durable and broad-spectrum resistance while the identified resistance loci will accelerate the breeding process. This will have far reaching implications on Striga management programs in SSA. Finally, our study revealed associated with *Striga* resistance in sorghum. This finding is directly applicable in sorghum improvement programs for *Striga* resistance using marker assisted selection, genetic modification or modern gene-editing technologies.

## Methods

### Plant material

We used 206 sorghum genotypes of the Generation Challenge Program sorghum reference set (RS) (www.icrisat.org/what-we-do/crops/sorghum/Sorghum_Reference.htm). The collection was originally obtained from the International Crops Research Institute for the Semi-Arid Tropics (ICRISAT) through their Nairobi, Kenya office. The organization maintains a vast repository for world sorghum that is well characterized and documented for ease of retrieval [42]. Detailed information on these genotypes is shown in (Table S1). The collection has been genotyped by sequencing [43] and SNP data is available at http://www.morrislab.org/data. This study used seeds of *S. hermonthica* from Western Kenya (Kisumu) located at 0.0699°S, 34.8169°E (Kibos isolate) harvested in 2012 and prepared as follows: *Striga* seed heads were collected from sorghum-infested farmer fields following approval and in accordance to regulations set by National Commission for Science, Technology and Innovation (NACOSTI). Harvested material was dried inside paper bags for 14 days. Heads containing *Striga* seeds were then threshed by lightly tapping the papers, and seeds separated from debris by passing through sieves with 250 and 150-μm openings as previously described [44].

### *Striga* seed conditioning

*Striga* seeds (25 mg) were surface sterilized using 10% commercial bleach (v/v) containing sodium hypochlorite for 10 min, followed by rinsing three times with sterile distilled water. The seeds were spread on filter papers (Whatman, GFA) placed inside a 90 mm Petri dish, then 5 ml of sterile distilled water added to the plates. Plates were sealed with parafilm, wrapped in aluminum foil and incubated at 28 °C for 14 days. To induce germination, 3 ml of filter-sterilized 0.1 ppm GR24 (Chiralix, Amsterdam) was added and seeds incubated for 12 h at 28 °*C. Striga* seed viability was determined using a microscope (Leica MZ7F; Leica, Germany), and only plates with more than 70% efficiency used in infection of sorghum roots.

### Sorghum growth and infection with *Striga*

We used a soil-free system, based on rhizotrons, to screen the sorghum lines for post-attachement resistance to *Striga* as previously described [16]. In this assay, resistance is measured by analyzing the mean number, lengths and total biomass of *Striga* seedlings attached on a host root. Successful colonization of a genotype by numerous and long *Striga* plants with a large biomass is interpreted as a susceptible response. In contrast, a resistance response is indicated by few, and short parasite seedlings with low *Striga* biomass.

The rhizotron assay was set up as follows: Sorghum seeds were germinated in plastic pots (10 × 10 × 7 cm) filled with vermiculite and watered with Long Ashton nutrient media [45]. Upon germination, seedlings were transferred to rhizotrons made from Petri plates measuring 25 × 25 × 5 cm (Nunc, Thermo Fisher Scientific, UK) prepared as follows: The base of the Petri plates was filled with vermiculite and the bottom lined with strips (25 × 4 cm) of high density foam to absorb excess water. The plates were overlaid with a 50 μm-thick nylon mesh to separate vermiculite and plant roots, but allow access to nutrients. The lid was replaced and secured with insulating tape. The chambers were then wrapped with aluminium foil and maintained inside a glasshouse under a 12-h light/12-h dark photoperiod, 60% humidity with day and night temperatures of 28 and 24 °C for 10 days. During this period, the plants were drip-fed with Long Ashton plant nutrient media [45]. To infect sorghum roots with *Striga*, rhizotrons were opened and sorghum roots carefully aligned with ~ 5000 pre-germinated *Striga* seeds using a soft paint brush. After infection, the chambers were closed, wrapped in aluminum foil and maintained in the glasshouse as described above. Five plants per genotype were screened in a randomized complete block design (RCBD) in three replicates.

### Analysis of post-germination resistance of sorghum against *Striga*

To identify sorghum genotypes resistant to *S. hermonthica*, we analyzed 3 metrics; number of *Striga* attachments on a host plant, length of the attached parasite seedlings and their biomass 21 days after infection (DAI). *Striga* seedlings attached to each host were harvested, placed in 90-mm Petri plates and photographed. We then used Image analysis software, ImageJ v.1.45 (http://rsb.info.nih.gov/ij) to determine length as well as the number of *Striga* seedlings per host plant. In addition, we determined *Striga* biomass after oven-drying the seedlings at 45 °C for 7 days. We generated means and standard deviations for all three metrics, then carried out analysis of variance (ANOVA) using statistical analysis software (SAS v. 9.1, SAS Institute, Cary, NC, USA) for comparisons across genotypes. We then used the Tukey’s honest significant difference (HSD) test (*p* ≤ 0.05) for mean separations and to assign groups to the genotypes based on resistance to *Striga*. Means of resistance for the top 50 resistant varieties for each metric were displayed as dot plots generated using the ggdotchart function in R.

To rank the resistance of sorghum genotypes in the diversity panel relative to known resistance controls i.e. IS9830 (an advanced cultivar of Caudatum race from East Africa) and an Indian Durra (N13, also annotated as IS18331 in some literature) we generated 5 categories of resistance. Genotypes with similar or significantly higher resistance than either N13 or IS9830 were grouped as “Highly Resistant”, while those with one mean separation group less resistance than either IS9830 or N13 were considered “Resistant”. Subsequent genotypes were categorized as “Moderately Resistant”, “Susceptible” or “Highly Susceptible” based on decreasing classes on mean separations. Ochuti, a *Striga*-susceptible farmer-preferred landrace was used as a susceptible check. To better understand the metric that provided the best resolution of resistance variation among the sorghum genotypes, we subjected the 3 metrics to PC analysis.

To further determine the resistance response of sorghum accessions using the combined metrics of number of *Striga* attachments, length and biomass, we used the Rank Summation Index (RSI) originally described by described by [20] but modified by [11]. In the modification, accessions with high *Striga* resistance (low number of attachments, short length and low biomass) were assigned lower ranks while more susceptible ones were given lower ranks. The resulting RSI scores were used to produce a heatmap for the highest ranking 50 accessions using the R package pheatmap.

### Analysis of mechanisms of post-germination resistance against *Striga*

Mechanisms of *Striga* resistance were evaluated in 20 accessions that displayed the lowest number of *Striga* attachments, the resistant controls (N13 and IS9830) and a highly susceptible accession (IS18829). These were done by analyzing the host-parasite interface, 9 days after infection (9DAI) through histological analysis. To achieve this, small sections of sorghum roots infected with *S. hermonthica* were excised, fixed in Carnoy’s fixative (4:1, 100% ethanol:acetic acid) and stained with 1% safranin in 30% ethanol for 5 min. Tissues were then cleared with choral hydrate (2.5 g/ml) for 12 h and the extent of parasite infection on the host roots documented using a Leica stereomicroscope MZ10F fitted with DFC 310FX camera.

Fixed tissue were then embedded by firstly pre-infiltrating them in 1:1 parts Technovit® solution (Haraeus Kulzer GmbH, Germany) and absolute ethanol for 2 h followed by infiltration in 100% Technovit® for 15 min. The tissues were transferred to a fresh Technovit® solution and maintained for 3 days. To embed, tissue in upright position were placed in 1.5 ml micro-centrifuge lids containing 1part Hardener® and 15 parts Technovit®. After setting, embedded tissues were mounted onto wooden blocks using the Technovit® 3040 kit following the manufacturer’s instructions (Haraeus Kulzer GmbH). For sectioning, we used the Leica RM 2145 microtome (Leica, Germany) to cut 5-μm-thick sections which were transferred onto glass slides dried on a hot plate at 65 °C for 30 min, stained using 0.1% toluidine blue O dye in 100 mM phosphate buffer for 2 min and washed in distilled water. Dry slides were overlaid with cover slips using DePex (BDH, Poole, UK), observed, and photographed using a Leica DM100 microscope fitted with a Leica MC190 HD camera, (Leica, Germany).

### Population structure of the sorghum diversity panel

To determine the hierarchical population structure of the sorghum reference panel, we subjected the SNP data to ADMIXTURE 1.3.0; a model-based software for estimation of ancestry in unrelated individuals using the maximum-likelihood method [46]. The analysis was performed for different clusters, referred to as K, ranging from 1 to 10 (for 10 replications per K). We then selected the most appropriate K-value based on the K that exhibited the lowest cross-validation error. To further elucidate genetic relationships among the sorghum germplasm, we converted the Hapmap genotype data, in Trait Analysis by aSSociation, Evolution and Linkage (TASSEL) to VCF format and used the resulting file to construct a neighbor-joining (NJ) tree using the Analyses of Phylogenetics and Evolution (APE) package in R [47]. To visualize the global origin of various sorghum genotypes, we utilized the Geographical Positioning System (GPS) coordinates available at https://www.morrislab.org/data [48] to construct a distribution map using the maptools package in R. Finally, we employed the principal component analysis (PCA), using ggplot2 package in R [49] to understand the scattering and genetic relatedness of the sorghum diversity panel under this study.

### Genome wide association study

Resistance data sets based on number of parasite attachments, length and biomass were used to identify SNPs associated with the resistance. Firstly, SNPs were filtered to only include those that had > 0.05 allele frequency leaving 247,975 SNPs and 204 genotypes. We then carried in a GWAS utilizing the Fixed and random model Circulating Probability Unification FarmCPU [50] algorithm implemented in the Genomic Association and Prediction Integrated Tool (GAPIT) as described in [51]. Kinship (K) was calculated using the default parameters. Quantile – quantile and Manhattan plots were both generated outputs of GAPIT analysis. We used Q-Q plots to verify control for population structure and appropriateness of the association model and Manhattan plots to visualize SNPs that were significantly associated with *Striga* resistance. The threshold of association (*p*-value) was determined for each trait using a false discovery rate (adjusted *p* < 0.05) in GAPIT. All significant SNP markers were mapped onto the *Sorghum bicolor* v3.1.1 genome [52] in Phytozome v12.1 [53] using JBrowse [54] based on physical positions obtained during SNP calling.

## Supplementary Information

**Additional file 1.**

**Additional file 2.**

**Additional file 3.**

**Additional file 4.**

## Data Availability

All data generated or analyzed during this study are included in this published article and its supplementary information files.

## Abbreviations

SSA: Sub-Saharan Africa
GWAS: Genome-wide association studies
GBS: Genotyping by sequencing
SNPs: Single Nucleotide Polymorphisms
Dmkr: Durra muskwaari
DC: Durra-caudatum
Gma: Guinea margaritiferum
RSI: Ranked summation index
NJ: Neighbour joining
CV: Cross Validation
PCA: Principal components analysis
DAI: Days after infection
HR: Hypersensitive reaction
FarmCPU: Fixed and random model Circulating Probability Unification
QQ: Quantile-Quantile
PDR: Pleiotropic drug resistance
ABC: ATP Binding Cassette
IFR: Isoflavon reductase
ERF: Ethylene-responsive transcription factor
JA: Jasmonic acid
SA: Salicylic acid
Prx: Peroxiredoxin
NERICA: New Rice for Africa
*SG3*: *Striga gesnerioides* race 3
QTL: Quantitative trait loci
RILs: Recombinant inbred lines
SAR: Systemic Acquired Resistance
ICRISAT: International Crops Research Institute for the Semi-Arid Tropics
RCBD: Randomized complete block design
ANOVA: Analysis of variance
HSD: Honest significant difference
TASSEL: Trait Analysis by aSSociation, Evolution and Linkage
GPS: Geographical Positioning System
GAPIT: Genomic Association and Prediction Integrated Tool
FDR: False Discovery Rate
NAC: NAM (No apical meristem)
ATAF: Arabidopsis transcription activation factor
CUC: Cup-shaped cotyledon
ABCG/PDR: ATP binding cassette G /Pleiotropic drug resistance
AP2/ERFs: APETALA 2/Ethylene Responsive Factors
APE: Analysis of Phylogenetics and Evolution
VCF: Variant Call Format
