# Supplementary material for: GWAS provides biological insights into mechanisms of the parasitic plant (Striga) resistance in sorghum
Source: BMC Plant Biol. 2021 Aug 21;21:392. doi: 10.1186/s12870-021-03155-7 (PMC8379865; doi:10.1186/s12870-021-03155-7)
Supplement: Supplementary file 1 — Additional file 1. [file 12870_2021_3155_MOESM1_ESM.docx]

**Supplementary information**

The following supplemental materials are available.

Additional file 1

Additional file 1: Figure S1. Principal component analysis (PCA) showing the distribution of sorghum accessions according to botanical races.

Additional file 2

Additional file 2: Figure S2. Levels of post-germination resistance to *S. hermonthica* in sorghum. Frequencies of *Striga* resistance in sorghum genotypes using **(A)** attachment, **(B)** length and **(C)** biomass). Bars represent percent genotypes (out of 206) under each resistance group categorized as follows; HR-highly resistant, MR-moderately resistant, R-resistant, S-susceptible, HS-highly susceptible. **(D)** Principal component analysis of the 206 sorghum genotypes based on resistance to *Striga* based on the aforementioned metrics.

Additional file 3

Additional file 3: Table S1. Excel file of sorghum accessions from the Generation Challenge Program GBS dataset. Only 206 sorghum accessions used in the study are presented [Submitted as a separate Excel file]

Additional file 4

Additional file 4: Table S2. Excel file of the resistance response of the 206 sorghum diversity panel accessions used in the study [Submitted as a separate Excel file]

Additional file 5

Additional file 5**:** Table S3. Excel file Top 50 most resistant accessions based on ranked summation index (RSI) of *Striga* attachments, *Striga* length, and *Striga* biomass [Submitted as a separate Excel file]

Additional file 1: Figure S1. Principal component analysis (PCA) showing the distribution of sorghum accessions according to botanical races. Inset is a scree plot showing the variance contributions of each component.


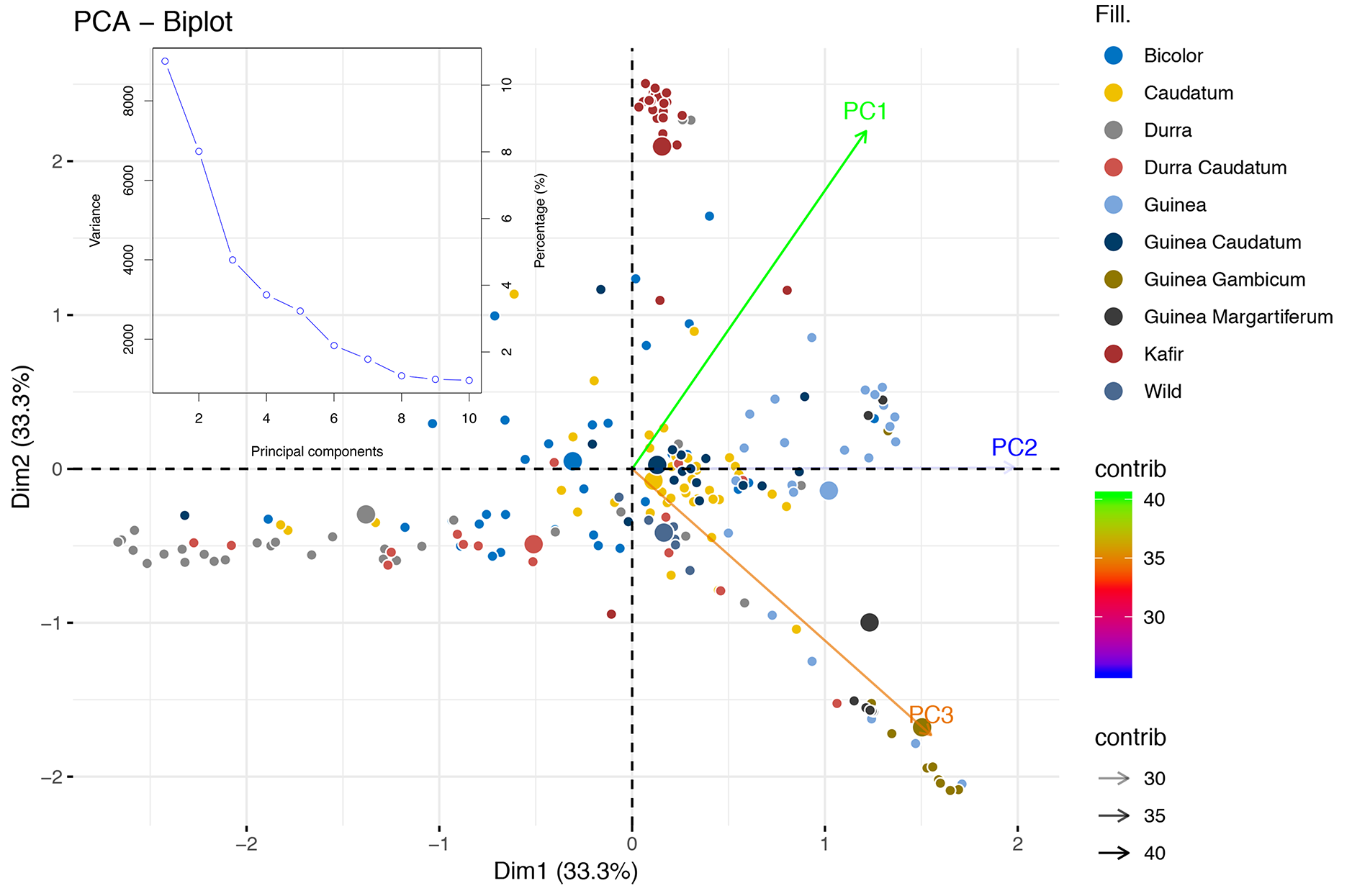


Additional file 2: Figure S2. Levels of post-germination resistance to *S. hermonthica* in sorghum. Frequencies of *Striga* resistance in sorghum genotypes using **(A)** attachment, **(B)** length and **(C)** biomass). Bars represent percent genotypes (out of 206) under each resistance group categorized as follows; HR-highly resistant, MR-moderately resistant, R-resistant, S-susceptible, HS-highly susceptible. **(D)** Principal component analysis of the 206 sorghum genotypes based on resistance to *Striga* based on the aforementioned metrics.

**
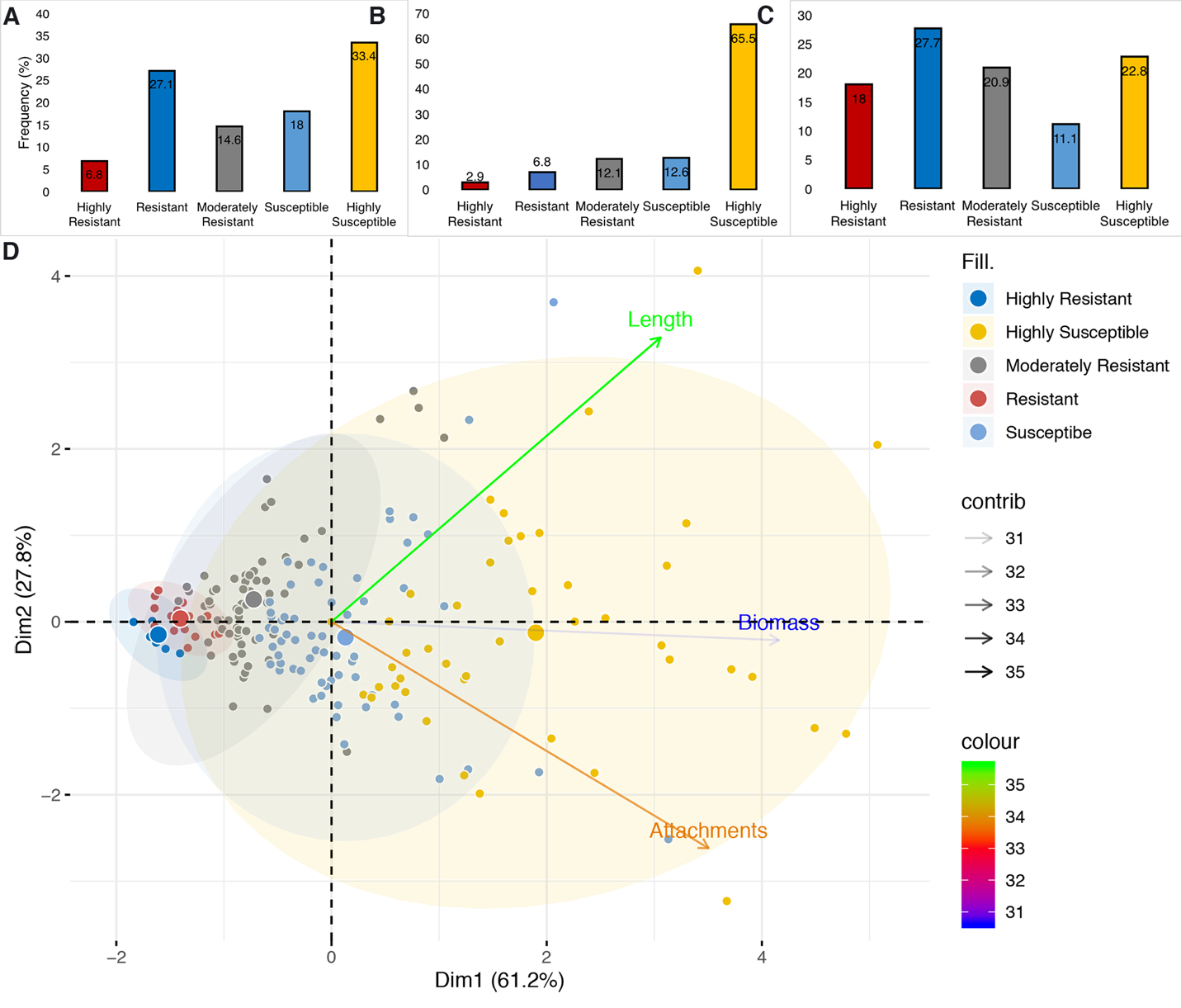
**
